# Supplementary material for: Inhibition of acetyl-CoA carboxylase by spirotetramat causes growth arrest and lipid depletion in nematodes
Source: Sci Rep. 2020 Jul 29;10:12710. doi: 10.1038/s41598-020-69624-5 (PMC7391777; doi:10.1038/s41598-020-69624-5)
Supplement: Supplementary file 1 — Supplementary Information. [file 41598_2020_69624_MOESM1_ESM.pdf]

Supplemental information

Inhibition of acetyl-CoA carboxylase by spirotetramat causes growth arrest and lipid depletion in nematodes

Philipp Gutbrod[1,2], Katharina Gutbrod[2], Ralf Nauen[3], Abdelnaser Elashry[1,4], Shahid Siddique[1,5], Jürgen Benting[3], Peter Dörmann[2], Florian M.W. Grundler[1]

[1] University of Bonn, INRES, Molecular Phytomedicine, Bonn, Germany, [2] University of Bonn, IMBIO, Molecular Physiology and Biotechnology of Plants, Bonn, Germany, [3] Bayer AG, Crop Science Division, Monheim, Germany, [4] current address: Strube Research GmbH & Co. KG, Schlansted, Germany, [5] current address: Dept. of Entomology and Nematology, UC Davis, US

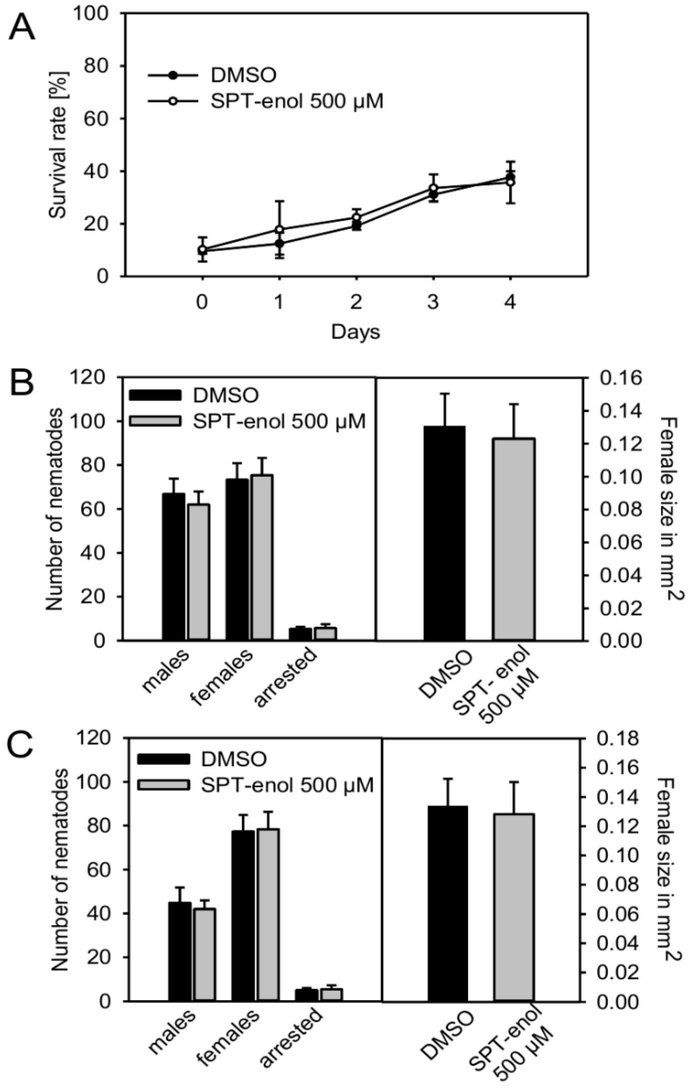

S1 Supplemental Figure 1: Effect of SPT-enol pre-incubation on J2 mortality and development of H. schachtii.

(A) Relative survival rate (%) of *H. schachtii* J2s larvae after treatment with 500 µM SPT-enol. *H. schachtii* J2 larvae were incubated in M9 buffer containing 500 µM SPT-enol or DMSO (control). The number of moving nematodes was counted daily. The data are given in average ± SD (n=5).

(B) Number of nematodes and sizes of females after treatment with 500 µM SPT-enol. *H. schachtii* J2 larvae were incubated in M9 buffer containing 500 µM SPT-enol or DMSO (control) and transferred onto plants after 48 hours. The data are given in average ± SD (n=10 for nematode numbers and n=30 for female sizes).

(C) Number of nematodes and sizes of females after treatment with 500 µM SPT-enol and 50 mM octopamine. *H. schachtii* J2 larvae were incubated in M9 buffer containing 50 mM octopamine and 500 µM SPT-enol or DMSO (control) and transferred onto plants after 48 hours. The data are given in average ± SD (n=10 for nematode numbers and n=30 for female sizes).

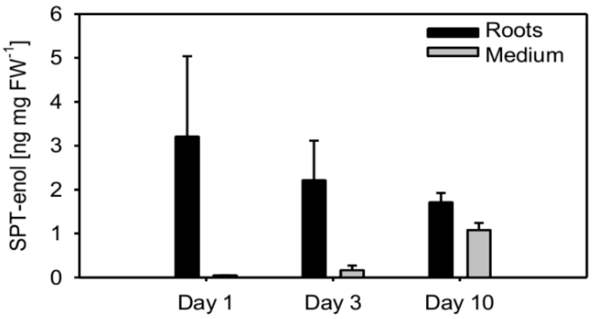

S2 Supplemental Figure 2 Content of SPT-enol in roots *of A. thaliana* and in the medium after foliar SPT application.

3 µL of SPT (200 mM) was applied foliarly onto the largest green leaf of 10-day-old *A. thaliana* plants. SPT-enol was quantified in roots of A. thaliana and in the medium after 1, 3 and 10 days. The data are given in average ± SD (n=4).

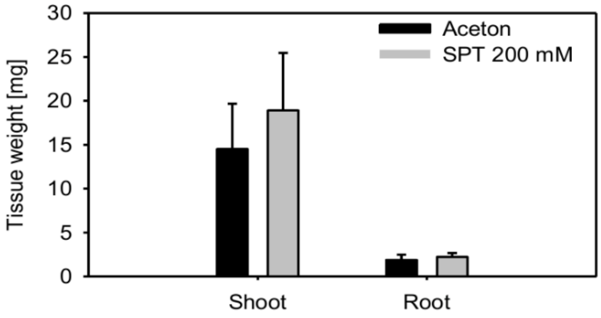

S3 Supplemental Figure 3 Effect of SPT on *A. thaliana* growth after foliar application.

3 µl of 200 mM SPT was applied onto the largest green leaf of 10-day-old *A. thaliana*. 10 days later, root and shoot weights were recorded (mg). No significant differences were found for root and shoot weight as compared to the control. The data are given in average ± SD (n=5).

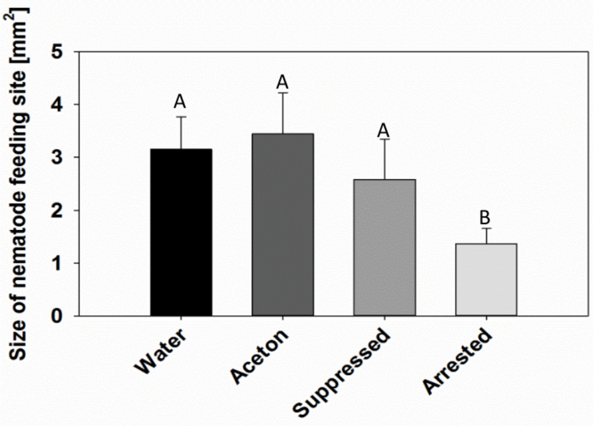

S4 Supplemental Figure 4: Concentration-dependent effect of SPT on development of nematode feeding site.

The sizes of nematode feeding sites were determined 14 DPI. The sizes of "suppressed" females are not significantly different as compared to the controls. Feeding sites of "arrested" nematodes are significantly smaller. The data are given in average ± SD. Anova $P < 0.001$, Tukey Test, N=30.

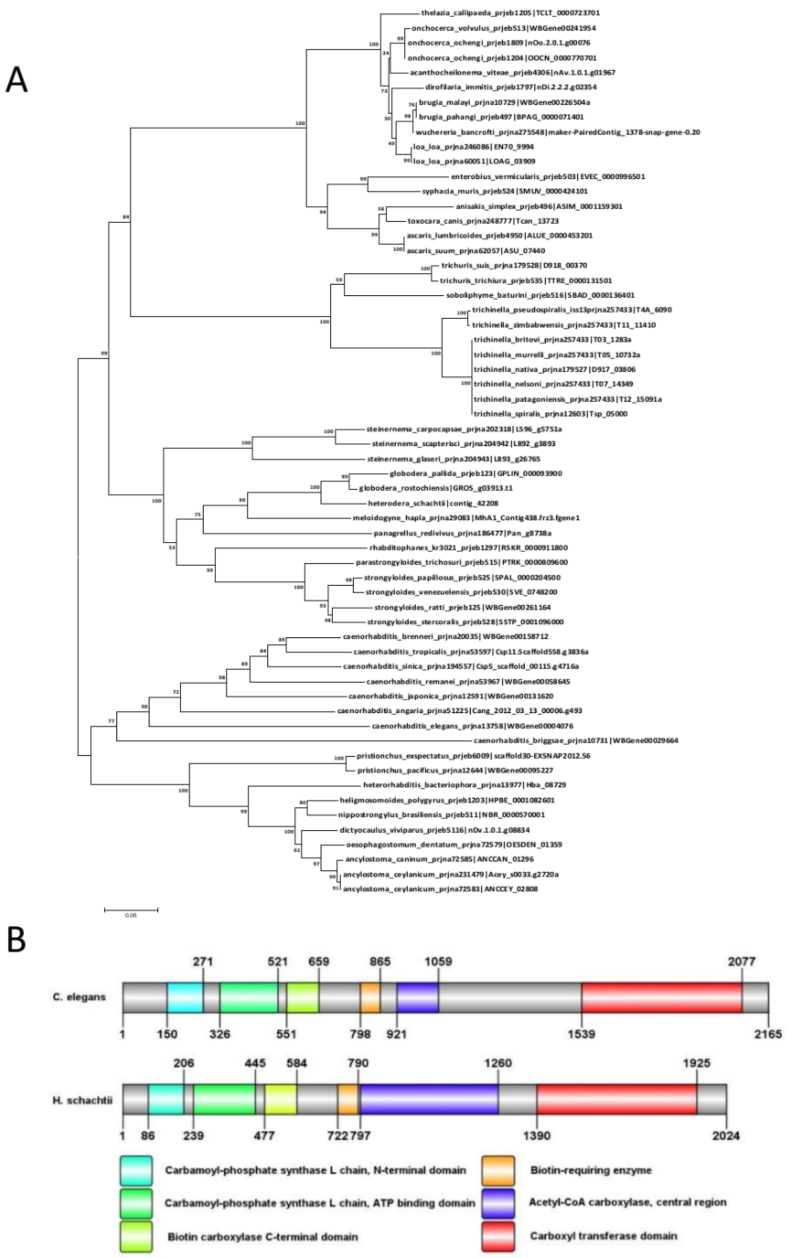

S5 Supplemental Figure 5: Sequence analysis of ACC from *H. schachtii*.

(A) Phylogenetic tree of ACC protein sequences of nematodes obtained from public databases. The mRNA sequence of the *C. elegans* POD-2 was used as query to find the corresponding *H. schachtii* ortholog. We found an mRNA contiq that contains an ORF coding for a putative protein with 2024 amino acids. This protein contains all functional domains required for ACC activity and is similar to other ACC sequences found in public databases. Protein alignment shows that HsACC is most similar to putative ACC´s of the cyst nematodes *Globodera pallida and Globodera rostochiensis*.

(B) Domain structure comparison of *C. elegans* and *H. schachtii* ACC. Domain structures were illustrated using DOG 2.0 (Ren et al. 2009).

Triacylglycerol	$[M+NH_4]^+$	Molecular Species
50:3	846.6612	16:1-17:cyclo-17:cyclo
51:3	860.6768	17:cyclo-17:cyclo-17:cyclo
52:3	874.6925	17:cyclo-17:cyclo-18:1
53:3	888.7081	17:cyclo-18:1-18:1, 17:cyclo-17:cyclo-19:cyclo
54:3	902.7238	18:1-18:1-18:1, 17:cyclo-18:1-19:cyclo
55:3	916.7394	18:1-18:1-19:cyclo, 17:cyclo-19:cyclo-19:cyclo

S6 Supplemental Table 1 Molecular species composition of TAGs in *C. elegans*.

The molecular species composition of TAGs was determined by scanning Q-TOF MS/MS spectra for neutral loss of fatty acyl-NH_3. The fatty acid composition of abundant molecular species is shown below.

**ACC-GW F**	GGGGACAAGTTTGTACAAAAAAGCAGGCTCTTTTTGCGAAAGGAATGACA
**ACC-GW R**	GGGGACCACTTTGTACAAGAAAGCTGGGTACTAATGTGCGGTGGGTATT
**ACC-T7 F**	TAATACGACTCACTATAGGGAGACTTTTTGCGAAAGGAATGACA
**ACC-T7 R**	TAATACGACTCACTATAGGGAGACACTAATGTGCGGTGGGTATT
**GFP-GW F**	GGGGACAAGTTTGTACAAAAAAGCAGGCTGATCCTGTTGACGAGGGTGT
**GFP-GW R**	GGGGACCACTTTGTACAAGAAAGCTGGGTTCAGTGGAGAGGGTGAAGGT
**GFP-T7 F**	TAATACGACTCACTATAGGGAGAGATCCTGTTGACGAGGGTGT
**GFP-T7 R**	TAATACGACTCACTATAGGGAGATCAGTGGAGAGGGTGAAGGT
**Actin-qPCR F**	CGTAGCACAACTTCTCCTTG
**Actin-qPCR R**	CGTGACCTCACTGACTACCT
**ACC-qPCR F**	CCAACTCCAAATCGCCAT
**ACC-qPCR R**	TCGCAGGACGGAAGCCTT

S7 Supplemental Table 2 Oligonucleotides used in this study.

Oligonucleotide names and sequences (5´-3´) are shown.
